# Long-Term Outcomes of Pediatric Cerebral Arteriovenous Malformations: A Ten-Year Single-Center Retrospective Study

**DOI:** 10.3390/medicina61071177

**Published:** 2025-06-29

**Authors:** Mei-Cheng Hsiao, Yuang-Seng Tsuei, Hung-Chuan Pan, Ming-Hsi Sun, Wen-Hsien Chen, Hung-Chieh Chen, Chiung-Chyi Shen, Chi-Ruei Li, Yu-Cheng Chou

**Affiliations:** 1 Department of Neurosurgery, Neurological Institute, Taichung Veterans General Hospital, Taichung 407, Taiwan; iaaron10251@gmail.com (M.-C.H.); astrocytoma2001@yahoo.com.tw (Y.-S.T.); hcpan2003@yahoo.com.tw (H.-C.P.); mhsun@vghtc.gov.tw (M.-H.S.); ccshen@vghtc.gov.tw (C.-C.S.); 2 Department of Post-Baccalaureate Medicine, College of Medicine, National Chung Hsing University, Taichung 402, Taiwan; chenws.tw@gmail.com; 3 Department of Neurological Surgery, Tri-Service General Hospital, National Defense Medical Center, Taipei 114, Taiwan; 4 Department of Medical Research, Taichung Veterans General Hospital, Taichung 407, Taiwan; 5 Department of Radiology, Taichung Veterans General Hospital, Taichung 407, Taiwan; hungchiehchen@gmail.com; 6 Department of Industrial Engineering and Enterprise Information, Tunghai University, Taichung 407, Taiwan; 7 School of Medicine, National Yang Ming Chiao Tung University, Taipei 112, Taiwan; 8 Department of Physical Therapy, Hungkuang University, Taichung 433, Taiwan; 9 Graduate Institute of Clinical Medicine, Chung Shan Medical University, Taichung 402, Taiwan; 10 Department of Applied Chemistry, National Chi Nan University, Nantou 545, Taiwan

**Keywords:** pediatric, cerebral, arteriovenous malformations, surgical resection, stereotactic radiosurgery, hydrocephalus

## Abstract

*Background and Objectives*: Pediatric cerebral arteriovenous malformations (AVMs) are associated with significant morbidity and mortality. The aim of this study was to assess the long-term outcomes of surgical excision and stereotactic radiosurgery (SRS) of cerebral AVMs in pediatric patients. *Materials and Methods*: A single-center retrospective analysis was conducted using data obtained from a single medical center between January 2012 and July 2022. The Modified Rankin Scale (mRS) at admission and discharge and the Spetzler–Martin (SM) scores were analyzed. *Results*: Among 45 patients (mean age 11.8 years), 19 patients (42.2%) received surgical resection, with good outcomes (mRS 0–2) in 16 patients and complete obliteration in all patients. In total, 26 patients (57.8%) were managed with SRS. After 36.3 months on average, complete obliteration in 19 of 26 patients (69.2%) was confirmed. Among the 7 SRS patients without complete obliteration, 6 had residual cerebral AVMs at the last follow-up, and 1 had recurrence. All patients receiving SRS had favorable outcomes (mRS 0–1) and no apparent radiosurgery-related complications. *Conclusions*: In our study, the surgical resection or SRS was selected based on individual patient conditions, and the overall outcomes were satisfactory. Both surgical resection and SRS proved to be effective treatment options. Microsurgical resection demonstrated a high rate of obliteration and remains a favorable therapeutic choice with acceptable risks for pediatric AVMs.

## 1. Introduction

Cerebral arteriovenous malformations (AVMs) are congenital vascular lesions characterized by a complex tangle of abnormal arteries and veins that are directly connected without an intervening capillary bed, resulting in arteriovenous shunting [1]. The overall hemorrhage rate of AVMs per year is 3.0%, with 2.2% for unruptured AVMs and 4.5% for ruptured AVMs. Once AVMs rupture, they cause intracranial hemorrhage (ICH), which can be life-threatening, with morbidity rates of 30–50% and mortality rates of 10–15% among afflicted patients [2,3].

Pediatric cerebral AVMs represent approximately 12–18% of all cerebral AVM cases [1]. A simplified risk model suggests that the likelihood of hemorrhage is roughly equivalent to 105 minus the patient’s age in years [4]. For instance, a 15-year-old with a cerebral AVM faces a 90% lifetime risk of a hemorrhagic event. Given the substantial cumulative risk and potential for life-long disability, early and appropriate intervention is a priority. Darsaut et al. [5] have suggested a reduced annual rupture risk of 3.2% following a confirmed obliteration. Moreover, Gross et al. have shown encouraging outcomes, with an overall annual hemorrhage rate of 0.3% and a recurrence rate of 0.9% following a multi-modal approach in treating pediatric cerebral AVMs [6].

Microsurgical obliteration is often the preferred method for treating low-grade AVMs, particularly when the lesion is located in a non-eloquent area [7,8]. However, for AVMs situated in deep or inaccessible locations, such as those in eloquent areas or classified as SM Grade III or higher, stereotactic radiosurgery (SRS) offers a safer and more effective alternative due to the increased surgical risks associated with these lesions [9,10,11,12,13]. SRS is also effective in pediatric cases, where the vascular morphology may be less mature than in adults, providing a comparable role in treatment [9,10,11].

In this retrospective study, we report the experience of our institution in managing both ruptured and unruptured pediatric cerebral AVMs over a period of ten years. To gain deeper insights into the clinical and functional outcomes of pediatric cerebral AVMs treated either through surgery or radiosurgery, we also conducted a review of the existing literature pertaining to these two distinct treatment approaches.

## 2. Methods

### 2.1. Study Groups

Data collected retrospectively from pediatric (<18 years of age at presentation) patients with ruptured or unruptured cerebral AVM who underwent interventional treatment in the Department of Neurosurgery, Neurological Institute, Taichung Veterans General Hospital, a medical center in Taiwan, between January 2012 and July 2022 were reviewed. To obtain more accurate results, we included patients who were fully treated with surgical resection or SRS and had complete clinical data both preoperatively and postoperatively. The exclusion criteria were patients who had expired, received transcatheter arterial embolization (TAE) with or without SRS, or were lost to follow-up.

The clinical data analysis included patient demographics (sex, age) and detailed symptomatic presentation (headaches, seizures, neurologic deficits). We retrospectively calculated the Modified Rankin Scale (mRS) at admission and discharge from hospital for all patients. For a post-treatment evaluation within three months, a DSA was the preferred imaging modality due to its superior resolution and dynamic flow information. However, when the DSA was not feasible due to availability or patient-specific factors, magnetic resonance angiography (MRA), a non-invasive alternative, was employed to assess angiographic obliteration and re-hemorrhage. The mRS scores, the presence of major or minor deficits related to treatment, re-hemorrhage, and ventriculo–peritoneal shunt (VPS) insertion were applied to retrospectively evaluate clinical outcomes at discharge from the hospital. Scores ranging from 0 to 2 indicated good clinical outcomes, while those with a score of 3–5 were considered disabled.

This retrospective study received approval from the Institutional Review Board of Taichung Veterans General Hospital, adhering to the ethical standards outlined in document No. CE17182A-5, and conformed to the guidelines governing human research ethics.

### 2.2. Statistical Analysis

A statistical analysis was performed using MedCalc software (version 14.12.0; Ostend, Belgium). Due to the skewed distribution of the SM scores observed upon visual inspection of histograms, a Mann–Whitney U test, a non-parametric test, was utilized to compare the scores between the surgery and SRS groups. A *p*-value of less than 0.05 was considered indicative of a statistically significant difference.

## 3. Results

**Figure 1:**
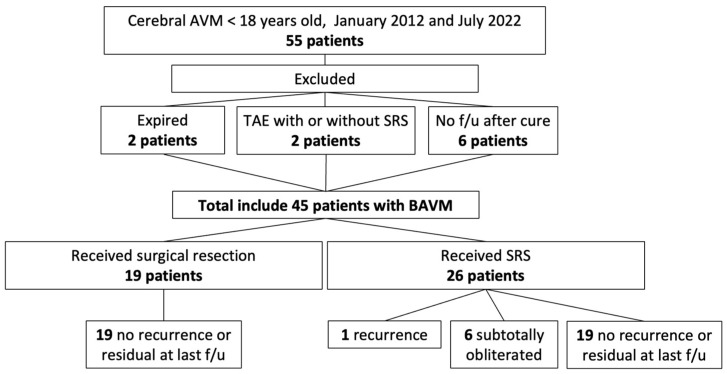
Flowchart of cohort selection of pediatric cerebral arteriovenous malformations. AVM = arteriovenous malformation; f/u = follow-up; SRS = stereotactic radiosurgery.

Between January 2012 and July 2022, 55 pediatric patients with cerebral AVMs were treated at our institution. After excluding 10 patients due to incomplete data, death, or loss to follow-up, 45 patients were included in the analysis (Figure 1). Of these, approximately 42% underwent surgical resection, while the remainder received the SRS. The baseline characteristics of the patients are summarized in Table 1. The cohort was predominantly male, with a mean age at diagnosis in the early teens. The most frequent initial presentation was consciousness disturbance, followed by seizures, with the majority of AVMs classified as low-grade (SM I–II).

Treatment outcomes are detailed in Table 2. At discharge, nearly all patients (97.8%) achieved good outcomes (mRS ≤ 2), with only one patient exhibiting neurological deficits (mRS 3–5). Complete obliteration was observed in all surgical cases and in approximately 69% of SRS cases after an average follow-up of over 3 years. VPS insertion was required in a small subset of patients, all with ruptured AVMs and higher-grade lesions (SM IV). In the SRS group, one patient experienced an asymptomatic recurrence, and several had subtotally obliterated lesions at the last follow-up. No major treatment-related complications (e.g., postoperative hemorrhage, infection, radiation necrosis) were noted beyond the need for VPS insertion in select cases.

**Figure 2:**
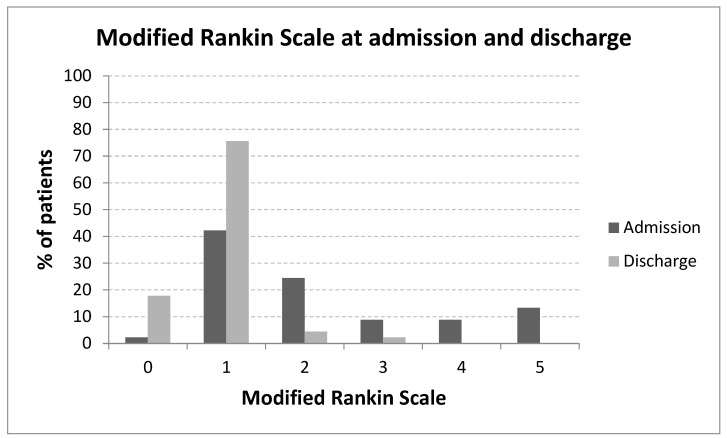
Relationship between mRS scores at admission and discharge.

**Figure 3:**
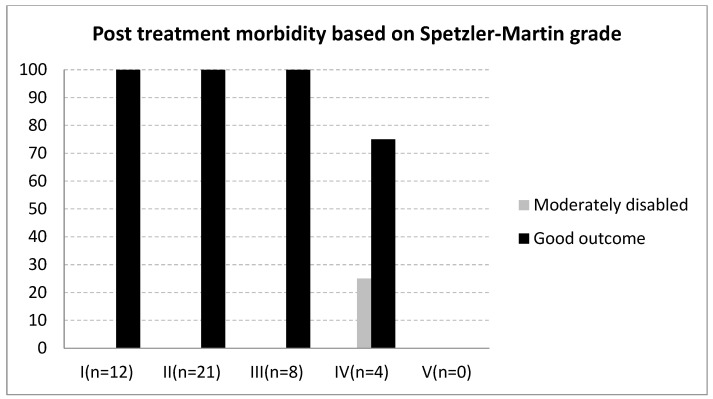
Post-treatment outcome based on Spetzler–Martin grade. Note: Moderately disabled was defined as Modified Rankin Score of 3; good outcome was defined as modified Rankin score of 0–2.

**Figure 4:**
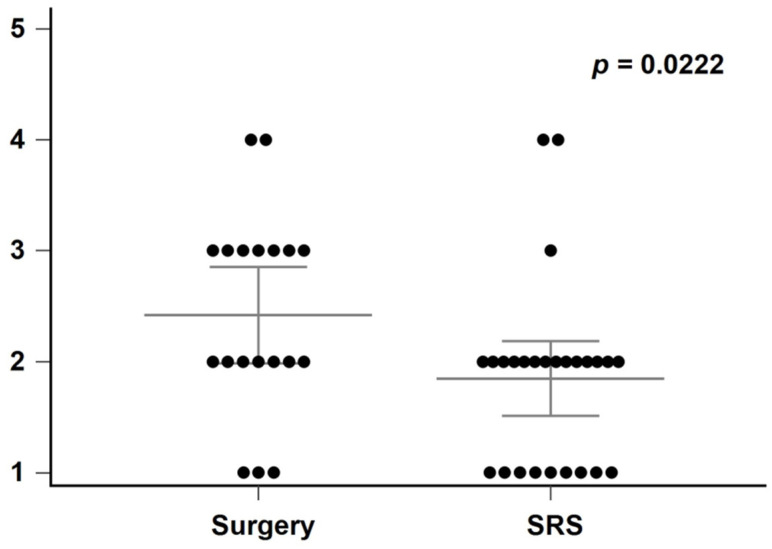
Comparison of SM grades between surgery and SRS.

The relationship between mRS scores at admission and discharge is illustrated in Figure 2, and post-treatment outcomes by SM grade are shown in Figure 3. A statistically significant difference in the SM grade distribution was observed between the surgery and SRS groups (*p* = 0.0222, Figure 4).

## 4. Discussion

Our study demonstrates predominantly positive outcomes in pediatric cerebral AVMs treatment, with a notable 97.8% of patients achieving an mRS score of 2 or less at discharge, following either the surgical resection or SRS (Figure 3). This is complemented by a low recurrence rate of 2.2% and minimal post-treatment neurological deficits, highlighting the effectiveness of tailored treatment strategies for these conditions. The favorable results observed in our study suggest that factors such as patient selection, AVM size, location, complexity, and quality of postoperative care play a critical role in treatment outcomes. While the exact impacts of the evaluation are challenging to quantify, they are essential considerations in treatment planning. Our promising findings underscore the need for ongoing research and comparison with broader datasets to validate these results and to better understand the underlying reasons for the observed differences in outcomes, which would allow for further refinement of the treatment strategies for pediatric cerebral AVMs.

Generally, higher SM scores are negatively correlated with the likelihood of cure [14]. We utilize the SM scores to distinguish patients who may benefit from a surgical intervention from those who would be better suited to other management strategies. Low-grade cerebral AVMs (SM I–III) may be treated via surgical resection, with a high cure rate, low morbidity, low mortality rates, and an immediate angiographic cure rate exceeding 80% [15]. However, the decision to select a treatment strategy for a pediatric cerebral AVM depends on its characteristics and the patient’s condition. In our series, surgical treatment and SRS were used for 17 and 24 patients, respectively, with low-grade AVMs (SM I–III). More families chose the SRS for their children with low-grade AVMs. Higher SM grades of AVM were mainly treated with surgery compared to SRS (*p* = 0.0222) (Figure 4) in our series.

In our patient cohort, surgical treatment was administered to 17 patients with low-grade AVMs (SM Grades I–III) and two patients with high-grade lesions (SM Grade IV), yielding favorable outcomes. Specifically, 16 patients achieved mRS scores of 0–2, with complete obliteration noted in all cases. Conversely, for cerebral AVMs located in eloquent regions or those that were low-grade but surgically inaccessible, the SRS was employed. According to the existing literature, the SRS provides an obliteration rate of up to 70%, with a 5% risk of complications [16]. In comparison, a study by Yen et al. [17] observed that 49.5% of pediatric patients achieved total angiographic obliteration two years following the SRS. Another study focusing on pediatric patients reported complete obliteration in approximately 65.9% of cases after a single-session SRS, with an overall complication rate, including new hemorrhage and neurological deficits, of 8.0% [18]. In our series, the SRS was used for 24 patients with low-grade AVMs (SM I–III) and 2 with high-grade lesions (SM IV) in eloquent areas, resulting in complete obliteration in 19 patients (69.2%) within an average of 36.3 months post-SRS, and no complications occurred. These comparative insights reveal that our results align closely with other studies in terms of obliteration rates. However, our study demonstrates a slightly higher success rate and a lower complication rate, underscoring the effectiveness of our treatment approach for pediatric cerebral AVMs.

Cerebral AVMs’ recurrence after an SRS-induced complete obliteration is rare, with annual risks of less than 1% [19,20]. The mechanism of recurrence after the SRS is unclear. However, several hypotheses have been proposed. At the forefront of these mechanisms is the possibility of incomplete obliteration. Despite imaging studies indicating total eradication, microscopic vestiges of the AVM network may persist. Over time, these residual vessels can potentially regenerate, leading to recurrence. Another compelling factor is neovascularization. The body’s healing response to radiation damage might inadvertently trigger the formation of new blood vessels. An overactivity of vascular endothelial growth factor could evolve into a new AVM, as the newly formed vascular networks mimic the abnormal structure and function of the original malformation [20,21,22,23]. Therefore, a long-term neuroimaging follow-up of patients with radiological evidence of complete AVM obliteration is recommended. In our study, we observed one patient with an AVM over the left corpus callosum who, after complete obliteration post-SRS, experienced a recurrence without any symptoms. Owing to the patient’s stable condition, we opted for regular follow-up to monitor neurological status and radiological findings.

In a study of 2093 AVM patients treated with the SRS, Yen et al. [24] found 159 patients had subtotally obliterated lesions, representing an incidence of 7.6%. These lesions, characterized by a visible draining vein but no visible nidus, did not result in any cases of bleeding post-diagnosis. Remarkably, without further treatment, 73.3% of these patients eventually achieved complete obliteration. This suggests that the SRS not only offers a reasonable chance of AVM obliteration but also that subtotal obliteration significantly lowers the risk of rebleeding. The necessity for retreatment in cases of subtotal obliteration is questionable, considering the substantial rate of spontaneous obliteration observed.

Regarding microsurgery for pediatric intracranial AVMs treatment, Bristol et al. [25] reviewed 82 children with AVMs treated surgically and reported a long-term obliteration rate of 90%. The perioperative mortality rate was 3.7%, and 81% of patients had excellent outcomes. Interestingly, 17% of patients who presented with hemorrhage had fair or poor outcomes, and the recurrence rate was 5.6%. In comparison, our findings were more favorable than those reported in the literature. Our long-term obliteration rate was 100%. No recurrence was found during the most recent follow-up. This discrepancy could be attributed to factors such as surgical techniques, patient selection, lesion characteristics, and postoperative care.

In children, ruptured cerebral AVMs are the major cause of ICH and intraventricular hemorrhage (IVH) with subsequent blockage of the arachnoid villi or cisterns resulting in hydrocephalus [26]. In the present study of children with cerebral ruptured AVM, the rate of VPS insertion was 4.4% [27,28]. However, recent theories, such as the Oreskovic–Bulat–Klarica theory, suggest that hydrostatic pressure differences and other mechanisms may also contribute to the development of hydrocephalus in these cases [29,30]. Our data revealed that children who needed VPS placement were more frequently disabled, with acute symptoms of hydrocephalus. Previous reviews have reported that shunt procedures can obtain an effective and successful management of hydrocephalus in an acute phase in patients with neurological deficits [31]. In our study, VPSs were placed in 4 (8.9%) of 45 patients with a moderate to severe degree of hydrocephalus. All these four patients had ruptured AVMs with high-grade lesions (SM IV). The small number of patients might be the reason why our VPS insertion rate was higher than in other studies. The occurrence of acute hydrocephalus following the rupture of a pediatric AVM can be influenced by several factors, including a low initial Glasgow Coma Scale score, the presence of IVH, and higher modified Graeb Scale (mGS) scores. It was observed that children who required a VPS had an initial hydrocephalus, necessitating an external ventricular drain, and tended to have higher mGS scores. Several studies also indicate that both IVH and subarachnoid hemorrhage (SAH) are significant factors in the development of acute hydrocephalus following the rupture of pediatric AVMs. The presence of the IVH seems to be particularly associated with the need for interventions like EVD and VPS, while SAH also contributes to the risk of hydrocephalus [23,27,32].

There are limitations to this study. A crucial limitation in comparing the efficacy of surgery and SRS for AVM treatment arises from the differences in indication, particularly the SM scores. Since our study focused on a single-modality treatment, making subgroup comparisons between surgical and SRS outcomes was challenging. Another significant limitation of our study was the relatively small number of patients. This factor, coupled with the retrospective and non-randomized nature of the study, poses certain constraints on the generalizability and robustness of our findings. Retrospective analyses, while valuable, may not capture the full spectrum of clinical variables and outcomes compared to prospective, randomized studies. Consequently, our results should be interpreted with caution, and further studies will be needed to fully elucidate the long-term outcome of pediatric cerebral AVMs.

## 5. Conclusions

The primary goal in treating pediatric cerebral AVMs is the preservation of neurological function. Favorable outcomes can be achieved through careful assessment of each lesion’s unique characteristics and the use of an appropriate single-modality treatment, such as the surgical resection or SRS. Microsurgical resection, in particular, offers a viable option with acceptable risks in selected cases. Our study adds valuable real-world data from a single-center experience within a public health insurance system operating under resource constraints. This distinguishes our practice from many high-volume centers in other countries and may offer relevant insights for institutions facing similar limitations.

## Figures and Tables

**Table 1:** Clinical characteristics of 45 patients with pediatric cerebral AVMs.

Variable			Patients (n = 45)	Number of Patients (%)
Male			28	62.2
Female			17	37.8
Age in years at diagnosis			11.8	-
Follow-up time in years			6.4	-
Initial presentation				
	Consciousness disturbance		21	46.6
	Seizure		10	22.2
	Neurologic deficit		6	13.3
		Limbs weakness	3	6.7
		Unsteady gait	2	4.4
		Aphasia	1	2.2
	Headache		7	15.5
	Vomiting		1	2.2
Spetzler-Martin grade				
	I		12	26.6
	II		21	46.6
	III		8	17.8
	IV		4	8.8
	V		0	0

**Table 2:** Modified Rankin Scale at admission and discharge.

Modified Rankin Scale	Admission	Discharge
0	1 (2.2)	8 (17.8)
1	19 (42.2)	34 (75.6)
2	11 (24.4)	2 (4.4)
3	4 (8.8)	1 (2.2)
4	4 (8.8)	0
5	6 (13.3)	0

## Data Availability

Data are available upon reasonable request. The datasets used during the current study are available from the Taichung Veterans General Hospital; however, restrictions apply regarding the availability of these data, as they are not publicly available. However, the data are available from the corresponding author upon reasonable request and with permission from the Taichung Veterans General Hospital.
